# Salient region detection through salient and non-salient dictionaries

**DOI:** 10.1371/journal.pone.0213433

**Published:** 2019-03-28

**Authors:** Mian Muhammad Sadiq Fareed, Qi Chun, Gulnaz Ahmed, Adil Murtaza, Muhammad Rizwan Asif, Muhammad Zeeshan Fareed

**Affiliations:** 1 School of Electronic and Information Engineering, Xi’an Jiaotong University, Xi’an, China; 2 School of Management, Xi’an Jiaotong University, Xi’an, China; 3 School of Science, MOE Key Laboratory for Non-equilibrium Synthesis and Modulation of Condensed Matter, State Key Laboratory for Mechanical Behaviour of Materials, Xi’an Jiaotong University, Xi’an, China; Huazhong University of Science and Technology, CHINA

## Abstract

Low-rank representation-based frameworks are becoming popular for the saliency and the object detection because of their easiness and simplicity. These frameworks only need global features to extract the salient objects while the local features are compromised. To deal with this issue, we regularize the low-rank representation through a local graph-regularization and a maximum mean-discrepancy regularization terms. Firstly, we introduce a novel feature space that is extracted by combining the four feature spaces like CIELab, RGB, HOG and LBP. Secondly, we combine a boundary metric, a candidate objectness metric and a candidate distance metric to compute the low-level saliency map. Thirdly, we extract salient and non-salient dictionaries from the low-level saliency. Finally, we regularize the low-rank representation through the Laplacian regularization term that saves the structural and geometrical features and using the mean discrepancy term that reduces the distribution divergence and connections among similar regions. The proposed model is tested against seven latest salient region detection methods using the precision-recall curve, receiver operating characteristics curve, F-measure and mean absolute error. The proposed model remains persistent in all the tests and outperformed against the selected models with higher precision value.

## Introduction

Salient Region Detection (SRD) is a procedure to confine the image according to the human visual attention and discovers the most useful and informative portion of an image. This procedure tries to approximate the possibility that the image region that is taking more attention comes out as a salient object. It is also a very helpful step because it is applied in many computer vision applications to reduce the computational complexity by only focusing on the salient parts of the image. The conventional saliency methods are separated into two groups as the bottom-up [1] and top-down [2]. The first category is a bottom-up method, which is a data-driven approach and it only depends on the prior knowledge of the object and the background. Whereas, the second category is a top-down approach, which is stimuli-driven and does not need prior information to detect the saliency.

**Fig 1:**
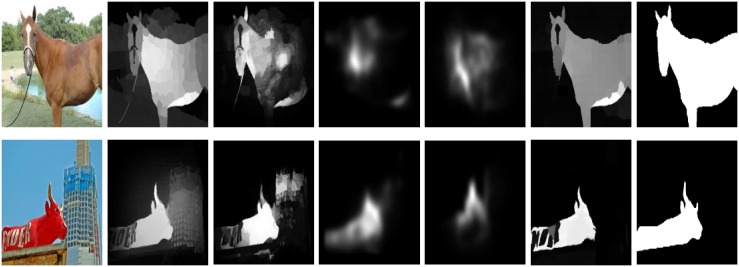
The reliability and accuracy of our designed method in the case, when the salient objects are touching marginally or completely to the image boundaries. The results are presented as: Input Image (II), MF [16], DS [6], DL [7], MT [8], the proposed model, and the Ground Truth (GT). The results are similar but not identical to the original images and is therefore for illustrative purposes only.

Existing techniques reveal the efforts of the researcher in finding the different features for SRD. The major portion of SRD literature is comprised of the bottom-up approaches [1], as these methods only consider low-level features and demonstrate a remarkable performance. Most of the approaches are only focusing on the visual features while ignoring the orientation and textural feature for computing the visual saliency [3–8]. Although, these schemes are successful in obtaining the visual saliency at some extent, however, these methods overlook some features like the orientation and textural feature, as only the visual features cannot capture all the image information [8–10]. The textural saliency is computed through textural features and very helpful for precisely capturing the pattern salient objects. Consequently, the visual and textural features are equally important for obtaining the precise saliency maps. Mainly, there are following issues with the current SRD models:

- The previously designed background dictionary-based methods [2, 11–14] used the limited information for the dictionary construction. Some methods collected the different color information [11], some engaged the boundary information [6, 15], a few of them utilized the center-remaining difference [13], and the remainder applied the center surrounded difference [16] for computing the background template. These methods are only focusing on the center part of the image. The background coefficient matrix compiled using the background priors are not satisfactory. As a result, the backgrounds parts embed with computed saliency and miss the accuracy or lose a lot of image information as shown in the second and the third column of Fig 1.
- The local methods compute the saliency by the rarity of neighbors or surrounded regions [9]. While the global methods extract saliency using the uniqueness of features over the entire scene [10]. The local features are efficiently captured through the local SRD methods but the global features are compromised. In the contrast, the global methods may easily attain all the global image information, and in this case, the local features are unnoticed as shown in the fourth and the fifth column of Fig 1. Hence, there is a need for a method, which equally treats all the local and global cues to compute SRD results.

To save the local and global image information, and to evade the unrelated background regions from embedding with the salient object, we exploit a Graph Regularize Term (GRT) and a Maximum Mean-discrepancy Regularization Term (MMRT). The GRT preserves the locality of the salient object and homogenously treats with all the parts of salient objects. While the MMRT decreases the distribution divergence between the similar regions. In this way, the background regions, which are mistakenly highlighted are suppressed by treating with MMRT. On the other hand, the salient object regions that are erroneously concealed during pre-processing are decorated again by exploiting this MMRT. In short, theses regularization terms transform the low-rank representation into a new appearance, which produces the saliency results more smoother, locally and globally homogeneous. The contributions of our designed method are given as:

- In this proposed framework more structural information of the image is incarcerated, for this purpose we concatenate four feature spaces to construct a new feature space that is consist of CIELab, RGB, HOG and LBP features.
- We propose a discriminative background dictionary that is constructed through the background and the foreground templates. This discriminative dictionary has more distinctive power because in our method the salient object is projected through the background and the foreground templates instead of the background template.
- We regularized the low-rank representation to save the similarity and locality of the regions. We introduced a GRT and a MMRT for the representation coefficients and the representation errors, respectively. By using, these terms the representation coefficients and the representation errors of similar regions contain similar saliency values when sparsely encoded with the discriminative dictionary.
- Our designed framework has more discerning power and more effective appearance as compared to the current low-rank representation-based methods because the low-rank representation is presented in a new way through the MMRT.

The remainder Sections of this paper are ordered in the following way: the existing schemes related to the SRD are discussed in the Section *II*. The details of our method like the feature extraction, the salient and non-salient template construction, and regularizing the low-rank representation through the Laplacian and mean discrepancy terms are given in the Section *III*. The evaluation metric, evaluation of our method, and its comparison with the state-of-the-art methods are given in Section *IV*. The conclusion and the future work are discussed in the Section *V*.

## 1 Related work and background

Several computational methods are proposed for SRD. The majority of the preceding schemes are appearance-based models, these models mainly depend upon the global or local contrast for their saliency map computation.

### 1.1 Dictionary learning-based SRD

The dictionary-based approaches [2, 11–14] facilitate in learning multifaceted labeling procedures and represent the image in a space where it can be easily processed. In [11], the basis vector is computed on the belief that the repeatedly activated bases contain less energy as compared to the rare bases. This model works selectively because the unpredicted bases are selected as salient clues. A dictionary for an image patch is constructed from a depository of natural images in [12]. Then, the sparse representation is utilized to find the contrast between each image patch. Shen et al., [13] optimize the objective of feature transformation and low-rank decomposition for training the dictionary. However, these methods manually trained their dictionaries using the top-down way. In [1, 14], the authors constructed the dictionary by only utilizing the center-surrounded patches without any training. However, the saliency results are not satisfactory because the inner-region of the salient object is not detected properly. In recent dictionary-based methods [6, 15], the author utilized the boundary information to extract the background dictionary. The saliency computed through this background dictionary is not clear because only the boundary information for background dictionary construction is insufficient. Currently, some methods engaged the center-remaining strategy [16], while other used the more background regions [17] to construct their background dictionary. However, most of the time the background templates contain limited information that leads to incorrect SRD.

### 1.2 Low rank representation-based SRD

Low-rank representation finds the lowest-rank from all the candidates that are available as a dictionary base. Low-rank representation works as an effective tool and computes all the global features information. On the other hand, the sparse representation seeks the sparsest representation of available data vector and computes all of the local features related to the salient objects. In [13], the authors optimize the objective of feature transformation and low-rank decomposition for training the dictionary. However, this method manually trained their dictionaries using the top-down way. The authors in [7] combined the low-rank representation and sparse representation to extract all the local and global features related to the salient object. However, due to the incompetent dictionary, the computed results are not persuasive. A salient object cannot be properly described by a single cue, it needs more cues to properly capture the salient object [8]. The authors generalized the low-rank representation as a multi-task sparsity pursuit and effectively combined the multiple features for salient object detection. However, all of the captured features are global and the saliency results are not as significant as expected. Dual low-rank pursuit [18] decomposes the image into a low-rank and a sparse part. This method uses the low-rank and sparse measures to characterize the global information and deals robustly with noises and background occlusions. However, this method remains unsuccessful in detecting the inner part of the salient object, as it is more emphasizing on the global image features.

### 1.3 Sparse representation-based SRD

The image boundary is always standing out as a part of the background. So, it can be very helpful in constructing the background template set [6, 15]. The authors computed the sparse representation error through this background template set. However, the computed results are not significant, when the salient object is touching the image boundary. The center-surrounded strategy is helpful in detecting, so, the authors in [16] engaged the center-remaining procedure to extract the dictionary. Then, the sparse reconstruction error is calculated through this dictionary. The computed saliency results averaged and improved through a multi-label inference process. To enhance the difference between the salient object and the background, a sparse coding-based generative model is discussed in [17]. To capture all information related to the image a superpixel sparse reconstruction-based model is defined in [3, 4]. However, the results generated by these models are not very clear because these methods only utilizing the local image information for SRD. Consequently, all these methods improved their results through an enhancement process, which recovers the lost information.

### 1.4 Global or local measures-based SRD

The previously designed SRD techniques are broadly divided into two categories local and global methods. The local methods compute the saliency by the rarity of neighbors or surrounded regions. While the global methods extract saliency using the uniqueness of features over the entire scene. In [14], the authors computed the saliency as the center-remaining difference of many features. Graph-based SRD method [9] exploits the rarity of different local features to compute the saliency map. A fuzzy growing approach is utilized to compute the saliency with the contrast of neighboring superpixels [19]. Ming Lin et al., [20] proposed the saliency of superpixels by incorporating the global features, namely spatial distribution and uniqueness. They used the PCA method to incorporate color and pattern distinctness to find the SRD. In [21], the authors computed the saliency by the global contrast between the image patches and their spatial position. They performed sampling based on the conventional three-color cues maps and PCA to extract the main features of the image patches. To extract a saliency map with high resolution that is dependent on color contrast, a Histogram Contrast (HC) method is defined in [10]. In [22], a non-local histogram approach is engaged to improve the efficiency of the method, and a smoothing procedure is applied to get rid of quantization artifacts. However, these proposed techniques are only suitable for simple natural images and lose their accuracy for highly patterned and textured images.

### 1.5 Multiple feature-based SRD

The existing approaches for SRD are mainly focusing on the color features, while ignoring the other features like texture, structure, and the orientation. Therefore, these types of methods are not successful when dealing with an image contains rich textural features. Many approaches for SRD use the RGB color model and few of them depending upon LAB or *YC*_*b*_*C*_*r*_ color space for their result calculation. The authors consider the near-infrared region with the RGB color model for SRD [23], as the near-infrared region provides more clues for recognition and categorization than the RGB color model. SRD using sparsity-based and graph-based model is proposed in [3], the authors combine the multi-features of colors with sparse representation model to compute the saliency. A method for SRD by combining multiple features of color distribution and contrast is proposed in [24], the authors exploited a multi-features color difference measure, a multi-features color distribution measure, and a multi-features salient object measure to compute the saliency. To exploit the multi-features constructing through image manifold of the different feature, a multi-feature enhancement procedure is discussed in [16]. However, these methods add some high contrast pixels with the salient object that lead to insignificant detection.

### 1.6 Foreground or background-based SRD

The discriminative schemes are also very important because these schemes help in enhancing the contrast between the background and foreground regions for SRD [24]. A number of discriminative strategies based models have appeared in current years. Shuang Li et al., [25] suggested that the saliency of a region is computed by the distance from the most assured background and foreground seeds. Hongyang Li et al., [26] proposed that the saliency of an object is estimated through propagating the cues extracted mainly from the certain object regions and background. The graph-based methods can capture more grouping features in the scene with the graph likeness. Graph similarity typically controls the performance of a graph-based method [27]. Some of them used the semi-supervised learning to approximate the similarities by incorporating local-grouping features deduced from the whole image. The foreground represents appearance consistency and uniformity, while the background many times reveals global or local connectivity with each of the four image boundaries [5]. In [17], a two-stage saliency scheme is defined which is based on relevance to the given query. After that, they used the graph-based manifold ranking procedure to rank the foreground and background cues. However, if the contrast is very less between the foreground and the background the computed saliency results are not accurate. Furthermore, it is very difficult to choose the position and the number of salient queries because these cues are generated through the random walks on the graphs, especially for the images that contain, unlike salient objects.

### 1.7 Deep Convolutional Neural Networks-based SRD

Since Deep Convolutional Neural Networks (DCNN) based methods [28–30] are engaged for SRD a tremendous progress has been achieved because of the availability of large visual datasets and GPU computing resources. The development of deeper and larger DCNNs [28–30] that could automatically learn more and more powerful feature representations with multiple levels of abstraction from big data. Significant progress has been made in the past few years to boost the accuracy levels of SRD [28–30], but existing solutions often rely on computationally expensive feature representation and learning approaches, which are too slow for numerous applications. In addition to the opportunities they offer, the large visual datasets also lead to the challenge of scaling up while retaining the efficiency of learning approaches and representations for both handcrafted and deeply learned features. In addition, given sufficient amount of annotated visual data, some existing features, especially DCNN features [28–30], have been shown to yield high accuracy for visual recognition. However, there are many applications where only limited amounts of annotated training data can be available or collecting labeled training data is too expensive. Such applications impose great challenges to many existing features.

The proposed method is different from the current SRD models in the following ways: the proposed model utilizes the combination of different color and texture models to accurately detect the salient object, the discriminative dictionary of the proposed model is constructed using the local as well as the global information that encodes the salient object at its best level, and the low-rank representation is presented in a new way that has more effective representation than before.

## 2 Proposed SRD framework

**Fig 2:**
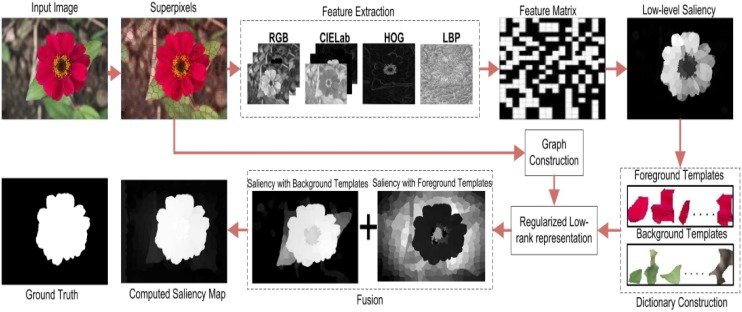
The pipeline of proposed SRD method.

The proposed model is discussed in detail in this section. First of all, the input image is over-segmented to extract the visual and textural features. In the second step, the boundary metric, candidate object metric, and candidate distance metric are combined to compute the low-level saliency that is later engaged to construct the salient and non-salient templates. At the final step, the low-rank representation is regularized through GRT and MMRT to compute the final saliency map as shown in Fig 2.

### 2.1 Visual and textural feature extraction

**Fig 3:**
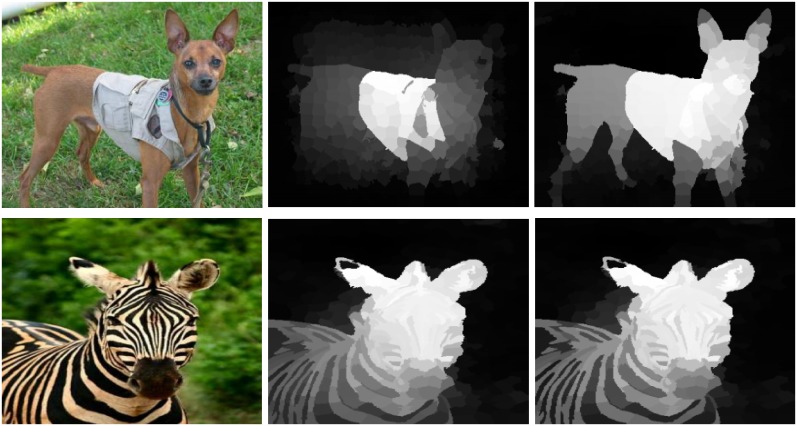
Importance of the visual as well as the textural features for computing a good saliency map is clear from the demonstrated results. The results demonstrated in the first row reveal that the results generated using one color model miss a lot of image information. However, the second row demonstrates that the saliency without the textural features is not significant. Therefore, both the visual and the textural features are equally important for computing the accurate saliency results. The results are similar but not identical to the original images and is therefore for illustrative purposes only.

To encode and capture the structural features of the given image, the given image is over-segmented to N superpixels through the SLIC [31]. SLIC adapts a k-means clustering approach to efficiently generate superpixels. Despite its simplicity, SLIC adheres to boundaries as well as or better than previous methods. At the same time, it is faster and more memory efficient, improves segmentation performance, and is straightforward to extend to super voxel generation. SLIC algorithm group pixels into perceptually meaningful atomic regions which can be used to replace the rigid structure of the pixel grid. Existing methods utilize the RGB color model or the CIELab space to compute the saliency. We also believe that a framework that is just utilizing visual features should not be capable of detecting the saliency persuasively as shown in Fig 3. However, we cannot deny that the major portion of an image is consisting of the visual features. To precisely capture the salient objects, all the visual and textural features are essential. Therefore, we combine the boundary, texture, geometry and spatial information to obtain our saliency results. Different attributes from four feature space are combined like (RGB, CIELab, HOG, LBP) to form a feature vector *f*_*i*_. The feature vectors are stacked in columns in feature matrix $F=\left[f_{i}^{1},\ldots .,f_{i}^{73}\right]\in R^{m\times N}$, where m represents the dimensions of the feature vector. The mean of the color feature is extracted from the superpixels, and we utilized after normalizing it. While the textural features like HOG and LBP feature are also extracted from the superpixels but after normalizing their histogram.

### 2.2 The discriminative dictionary

The discriminative dictionary is constructed using the local and the global features to encode salient object at its best level. We use the boundary metric, the candidate objectness metric, and the candidate distance metric to construct the dictionary. The details of these metrics are given in the next subsections.

#### 2.2.1 Boundary metric

Current SRD methods explicitly exploit the background features to compute their salient region map. As they believe that the background contrast, background connectivity, boundary information, and boundary connectivity information is obligatory for complete SRD. Since the salient object always appears near the center of the image and the boundary superpixels have possibly been the part of the background. We computed the distance of *i*^*th*^ superpixel from boundary superpixel *j*^*th*^ using the following expression:

$$\begin{array}{c} G{\left(i\right)}^{Bnd}=\frac{1}{B}\sum_{j=1}^{B}{∥f_{i}-f_{j}^{Bnd}∥}_{2}^{2} \end{array}$$ (1)

where, $f_{j}^{Bnd}$ is the boundary feature vector and B represents the number of background superpixels. The term $f_{i}-f_{j}^{Bnd}$ expresses the difference of the feature vector and utilized after normalizing it in the range [0, 1].

#### 2.2.2 Candidate objectness metric

To compute the candidate objectness, we computed a series of window priori that contains the probability of salient object and choose more than 3000 trial windows. The candidate objectness *C*_*Obj*_ map is computed after summing up these chosen samples. The candidate objectness metric for *i*^*th*^ superpixel is computed using the following expression:

$$\begin{array}{c} G{\left(i\right)}^{Obj}=\frac{1}{N(h_{i})}\sum_{a,b}C_{Obj}\left(a,b\right) \end{array}$$ (2)

where, the *N*(*h*_*i*_) is the number of pixels in *h*_*i*_ and (*a*, *b*) are the coordinates of the *h*_*i*_.

#### 2.2.3 Candidate distance metric

The candidate distance metric is very helpful in prominenting the salient object part. The candidate distance metric *G*(*i*) expression is given as:

$$\begin{array}{c} G{\left(i\right)}^{Dis}=\exp\left[-\left(\frac{x_{i}-x_{c}}{2\sigma_{x}^{2}}-\frac{y_{i}-y_{c}}{2\sigma_{y}^{2}}\right)\right] \end{array}$$ (3)

where, the *s*_*c*_ is the middle point of the salient object, and computed as:

$$\begin{array}{c} s_{c}=\{\begin{array}{c} x_{c}=\sum_{i,j=1}^{n}\frac{s_{i}}{\sum_{j=1}^{n}s_{j}}x_{i}\\ y_{c}=\sum_{i,j=1}^{n}\frac{s_{i}}{\sum_{j=1}^{n}s_{j}}y_{i} \end{array} \end{array}$$ (4)

where, *σ*_*x*_ = *x*_*c*_ and *σ*_*y*_ = *y*_*c*_ are the image center coordinates, *x*_*i*_ and *y*_*i*_ are the superpixel coordinates, *s*_*i*_ and *s*_*j*_ are the *i*^*th*^ and *j*^*th*^ superpixels of the image. Finally, the three low-level features are exploited to compute the low-level saliency map *SM*^*LL*^ of the *i*^*th*^ superpixel as:

$$\begin{array}{c} SM^{LL}\left(i\right)=G{\left(i\right)}^{Obj}\times G{\left(i\right)}^{Bnd}\times G{\left(i\right)}^{Dis} \end{array}$$ (5)

Here, we combined the low-level features to construct our dictionaries and the image representation is locally similar and smooth. The dictionary compiled on the basis of background template is not enough to compute the precise the saliency map. Therefore, we computed the salient and non-salient dictionaries (*D*^*ST*^, *D*^*NT*^) based on the *γ*_1_ and *γ*_2_ as:

$$\left\{ \begin{array}{c} D^{ST}=\left[f_{i}|SM^{LL}\left(i\right)\leq \gamma_{1}Mean\left(SM^{LL}\right)\right]\\ D^{NT}=\left[f_{j}|SM^{LL}\left(j\right)\geq \gamma_{2}Mean\left(SM^{LL}\right)\right] \end{array}\right.$$ (6)

**Fig 4:**
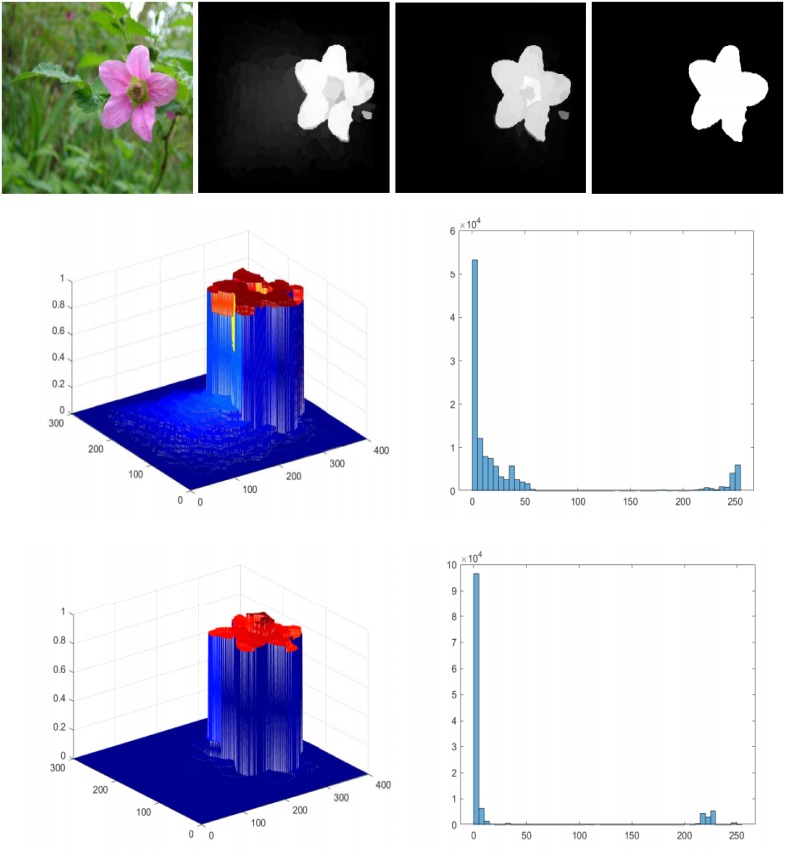
Importance of proposed discriminative dictionary for computing accurate saliency map is obvious from the demonstrated results.

The values of parameters *γ*_1_ = 0.35 and *γ*_2_ = 1.05 are set according to the experiments. The current dictionary-based SRD methods collect the boundary information [6, 15], the center-remaining difference [13], and center surrounded difference [16] for constructing the dictionary. Their performance remains satisfactory if the salient objects stay in the center of the scene. These methods have poor performance when the salient objects pop out near the image boundaries. Keeping in mind this issue, we designed a discriminative dictionary that is dependent on the salient object position. Not only this, we combine the boundary information, high-contrast background information, and the salient position information to compute the discriminative dictionary. The proposed discriminative dictionary is very helpful in salient object detection and very effective in suppressing the background part without disturbing the salient object as demonstrated in Fig 4.

### 2.3 Locality preservation method

In the recent literature of the SRD, sparse reconstruction based method are widely applied to detect the salient object and successively got the average results. They used the classical method without utilizing any global information related to the image. Therefore, sometimes it emphasizes the intensity of same regions and the obtained results have marginally less contrast as compared to the background regions. We exploited a GRT and a MMRT to preserve the local features and the global features related to the image and to evade the unrelated background from embedding with the salient object. The GRT preserves the local features of the salient object and homogenously treats with all the parts of salient objects. While the MMRT decreases the distribution divergence between the regions. In this way, the background regions, which are mistakenly highlighted are suppressed by treating with MMRT. On the other hand, the salient object regions that are erroneously concealed during pre-processing are decorated again by exploiting this MMRT. In short, theses regularization terms transform the low-rank representation into a new look, which has more smooth, locally and globally homogeneous. First, we exploited the following GRT:

$$\begin{array}{c} R\left(x\right)=\frac{1}{2}\sum_{i=1}^{n}\sum_{j=1}^{m}{∥z_{i}-z_{j}∥}_{2}^{2}w_{ij} \end{array}$$ (7)

$$\begin{array}{c} =tr(ZLZ^{T}) \end{array}$$ (8)

where, *Z* = [*z*_1_, *z*_2_, *z*_3_, …, *z*_*N*_] ∈ *R*^*K*×*N*^ is a representation coefficient matrix, the affinity matrix *W* = *w*_*ij*_ is employed to compute the weights between the connected regions. The Laplacian matrix *L* is defined as *L* = *C* − *W*, here *C* is the diagonal degree matrix *C* ∈ *R*^*N*×*N*^, which is $C_{ii}=\sum_{j=1}^{n}W_{ij}$. We computed the weights using the color, LBP, and HOG features. As we discussed earlier, the more accurate results are computed through the visual and textural features. Therefore, we assigned more weights to visual features as the colors contain the major portion of the image structure. If the weights are assigned according to the above-discussed limitation, then the constructed graph is connected marginally. A major portion of the regions has zero weights because the similar segments contain the similar saliency values. So, we applied the *K* − *adjacent* graph model to fully utilized the visual and textural information. After exploiting the GRT with the low-rank representation [7] the expression can be written as:

$$\begin{array}{c} \min_{Z,E}{∥Z∥}_{1}+\alpha_{1}{∥E∥}_{2,1}+\alpha_{2}tr\left(ZLZ^{T}\right) \end{array}$$ (9)

$$\begin{array}{c} s.\;t.\;\;\;\;\;\;F=DZ+E \end{array}$$

where, *Z* ∈ *R*^*K*×*N*^ is a representation coefficient matrix, and *E* ∈ *R*^*m*×*N*^ is the reconstruction errors matrix to the input image *F* ∈ *R*^*m*×*N*^. While *D* is discriminative dictionary *D* ∈ *R*^*m*×*K*^. The combination of the basis vector of the discriminative dictionary is used to represent a superpixel. While the *α*_1_ and the *α*_2_ are Graph Regularization (GR) and sparsity parameters, respectively. The designed framework contains all the locality and adhesiveness of the background and the foreground features to obtain the sparse coefficients for all the probability distributions. Even though the K-adjacent neighbor model is utilized to construct the graph, if we fail to obtain all the intrinsic features for sparse reconstruction coefficient probability distributions, the distribution divergence between the same regions (from similar to similar and dissimilar to dissimilar) remain unchanged, which can affect the obtained results. To deal with this issue, there is a need to decrease the distribution divergence between the same regions of the foreground and the background part in low-rank representation. The appropriate distributions of sparse coefficients are accomplished by expressing the data points through the empirical maximum mean-discrepancy matrix [32], which is applied as a non-parametric distance measure to keep the balance between the similar regions. We compute the spatial distance between the consecutive regions (from similar to similar and dissimilar to dissimilar) using *K* − *dimensional* co-efficient as follows:

$$\begin{array}{c} \Omega \left(e\right)=∥\frac{1}{n_{r}}\sum_{i=1}^{n_{r}}e_{i}-\frac{1}{n_{u}}\sum_{j=n_{r}+1}^{n_{r}+n_{u}}e_{j}{∥}^{2} \end{array}$$ (10)

where, *n*_*r*_ and *n*_*u*_ are examples foreground and background regions.

$$\begin{array}{c} =\sum_{i=1}^{n}\sum_{j=1}^{m}e_{i}^{T}e_{i}M_{ij} \end{array}$$ (11)

$$\begin{array}{c} =tr(EME^{T}) \end{array}$$ (12)

where, *M* is computed as follows:

$$\begin{array}{c} M=\{\begin{array}{c} \frac{1}{n_{r}^{2}}\;\;\;\;\;\;\;e_{i},e_{j}\;\epsilon D_{r}\\ \frac{1}{n_{u}^{2}}\;\;\;\;\;\;\;e_{i},e_{j}\;\epsilon D_{u}\\ -\frac{1}{n_{r}n_{u}}\;\;\;\;\;\;\;Otherwise \end{array} \end{array}$$ (13)

where, $D_{r}=\left\{\left(e_{1}^{i},e_{2}^{j}\right),....,\left(e_{r_{n}}^{i},e_{r_{n}}^{j}\right)\right\}$ and $D_{r}=\left\{\left(e_{1}^{i},e_{2}^{j}\right),....,\left(e_{r_{n}}^{i},e_{r_{n}}^{j}\right)\right\}$ are the foreground regions and background regions, respectively. After exploiting this regularization term, the discriminative dictionary is constructed and the distribution divergence from similar to similar and dissimilar to dissimilar regions is decreased by developing the *Z*. The following function is achieved after regularizing the low-rank representation term as:

$$\begin{array}{c} \min_{Z,E}{∥Z∥}_{1}+\alpha_{1}{∥E∥}_{2,1}+\alpha_{2}tr\left(ZLZ^{T}\right)+\alpha_{3}tr\left(EME^{T}\right) \end{array}$$ (14)

$$\begin{array}{c} s.\;t.\;\;\;\;\;\;F=DZ+E \end{array}$$

**Fig 5:**
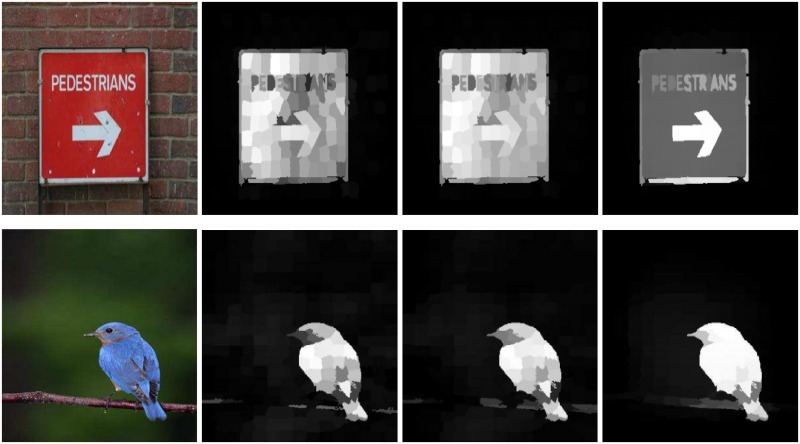
Comparison between different steps of our model and effectiveness of regularization terms. The results are arranged as: II, low-rank representation saliency map, our GRT map, and our MMRT map.

After exploiting the GRT and MMRT to the low-rank representation, it is transferred to a new appearance that has a more effective representation as shown in Fig 5.

## 3 Optimization

The above-discussed optimization problem is convex, this optimization is solved through [33, 34] and we also need to minimize this augmented Lagrangian function:

$$\begin{array}{c} \begin{array}{c} L\left(Z,E,Y\right)={∥Z∥}_{1}+\alpha_{1}{∥E∥}_{2,1}+\alpha_{2}tr\left(ZLZ^{T}\right)+\alpha_{3}tr\left(EME^{T}\right)\\ +<Y,F-DZ-E>+\frac{\mu }{2}{∥F-DZ-E)∥}_{F}^{2} \end{array} \end{array}$$ (15)

where, *μ* and *Y* are penalty parameter and Lagrangian multiplier, respectively. The notation <⋅> used to indicate the inner-product of two-matrices. This unconstrained problem is to minimize through *Z* and *E*.

### 3.1 Update Z

$$\begin{array}{c} Z=\arg\min_{Z}{∥Z∥}_{1}+\alpha_{2}tr\left(ZLZ^{T}\right)+<Y,F-DZ-E>+\frac{\mu }{2}{∥F-DZ-E∥}_{F}^{2} \end{array}$$ (16)

$$\begin{array}{c} Z=\min_{Z}{∥Z∥}_{1}+\alpha_{2}tr\left(ZLZ^{T}\right)+\frac{\mu }{2}{∥F-DZ-E+\frac{1}{\mu }Y∥}_{F}^{2} \end{array}$$ (17)

$$\begin{array}{c} Z=\arg\min_{Z}{∥Z∥}_{1}+g\left(Z\right) \end{array}$$ (18)

where, $g\left(Z\right)=\alpha_{2}tr\left(ZLZ^{T}\right)+\frac{\mu }{2}{∥F-DZ-E+\frac{1}{\mu }Y∥}_{F}^{2}$, the solution of above equation can be found through [33, 34] as:

$$\begin{array}{c} Z^{\left(t+1\right)}=\arg\min_{Z}\frac{1}{\eta^{\left(t\right)}}{∥Z∥}_{1}+\frac{1}{2}{∥Z-(Z^{t}-\frac{1}{\eta^{\left(t\right)}}\nabla_{z}g\left(Z^{\left(t\right)}\right))∥}_{F}^{2} \end{array}$$ (19)

$$\begin{array}{c} \eta^{\left(t\right)}=1.02(2\alpha_{2}{∥L∥}_{F}^{2}+\mu^{\left(t\right)}{∥D^{T}D∥}_{F}^{2}) \end{array}$$ (20)

where, ∇_2_ *g*(*Z*^(*t*)^) is partial-differential *g*(*Z*) w.r.t. Z and is calculated as:

$$\begin{array}{c} \nabla_{2}g\left(Z^{\left(t\right)}\right)=2\alpha_{2}Z^{\left(t\right)}L-\mu D^{\left(t\right)}(F-DZ^{\left(t\right)}-E^{\left(t\right)}+\frac{1}{\mu^{\left(t\right)}}Y^{\left(t\right)}) \end{array}$$ (21)

The above-defined equation’s solution is given as:

$$\begin{array}{c} Z^{\left(t+1\right)}=S_{\frac{1}{\eta_{Z}^{\left(t\right)}}}(Z\left(t\right)-\frac{1}{\eta_{2}^{\left(t\right)}}\nabla_{2}g\left(Z^{\left(t\right)}\right)) \end{array}$$ (22)

where, threshold function *S*_*τ*_(*w*) is defined as:

$$\begin{array}{c} S_{\tau }\left(w\right)=\{\begin{array}{c} w-\tau \;\;\;\;\;\;if\;w>\tau \\ w+\tau \;\;\;\;\;\;if\;w<\tau \\ 0\;\;\;\;\;\;Otherwise \end{array} \end{array}$$ (23)

### 3.2 Update E

$$\begin{array}{c} E=\arg\min_{E}\alpha_{1}{∥E∥}_{2,1}+\alpha_{3}tr\left(ELE^{T}\right)+<Y,F-DZ-E>+\frac{\mu }{2}{∥F-DZ-E∥}_{F}^{2} \end{array}$$ (24)

$$\begin{array}{c} E=\min_{E}\alpha_{1}{∥Z∥}_{2,1}+\alpha_{3}tr\left(ELE^{T}\right)+\frac{\mu }{2}{∥F-DZ-E+\frac{1}{\mu }Y∥}_{F}^{2} \end{array}$$ (25)

$$\begin{array}{c} Z=\arg\min_{E}\alpha_{1}{∥Z∥}_{2,1}+g\left(E\right) \end{array}$$ (26)

where, $g\left(E\right)=\alpha_{3}tr\left(ELE^{T}\right)+\frac{\mu }{2}{∥F-DZ-E+\frac{1}{\mu }Y∥}_{F}^{2}$, the solution of above equation can be found through [33, 34] as:

$$\begin{array}{c} E^{\left(t+1\right)}=\arg\min_{E}\frac{\alpha_{1}}{\eta_{E}^{\left(t\right)}}{∥E∥}_{2,1}+\frac{1}{2}{∥E-(E^{\left(t\right)}-\frac{1}{\eta_{E}^{\left(t+1\right)}}\nabla_{E}g\left(E^{\left(t\right)}\right))∥}_{F}^{2} \end{array}$$ (27)

$$\begin{array}{c} \eta_{E}^{\left(t\right)}=1.02(2\alpha_{3}{∥L∥}_{F}^{2}+\mu^{\left(t\right)}) \end{array}$$ (28)

where, ∇_2_*g*(*E*^(*t*)^) is partial-differential *g*(*E*) w.r.t. E and is calculated as:

$$\begin{array}{c} \nabla_{E}g\left(E^{\left(t\right)}\right)=2\alpha_{3}E^{\left(t\right)}L-\mu (F-DZ^{\left(t\right)}-E^{\left(t\right)}+\frac{1}{\mu }Y^{\left(t\right)}) \end{array}$$ (29)

The above-defined equation’s solution is given as:

$$E^{\left(t+1\right)}(:,i)=\{\begin{array}{ll} \frac{\left(\|H(:,i){\|}_{2}-\frac{\alpha_{1}}{\eta_{E}^{\left(t\right)}}\right)}{\|H(:,i){\|}_{2}}H(:,i) & if\|H(:,i){\|}_{2}\geq \frac{\alpha_{1}}{\eta_{E}^{\left(t\right)}}\\ 0 & Otherwise \end{array}$$ (30)

The complete summary is given in Algorithm 1 and the details of calculations are given below as:

**Algorithm 1** Solving Eq (17) through [33, 34]

1: **Input**: Feature matrix F, Parameter *α*_1_, *α*_2_, *α*_3_ and Laplacian matrix L

2: **Output**: E and Z

3: Initialize *E*^(0)^ = 0, *Z*^(0)^ = 0, *Y*^(0)^ = 0, *μ*^(0)^ = 1, *μ*_(*max*)_ = 10^(10)^, *ρ* = 1.1, *t* = 0, *ε*_1_ = 10^−^3, *ε*_1_ = 10^−^6

4: Repeat

5: Fix E and update using Eq (22)

6: Fix Z and update using Eq (30)

7: Update the Multiplier Y: *Y*^(*t*+1)^ = *Y*^(*t*)^ + *μ*^(*t*)^(*F* − *DZ*^(*t*)^ − *E*^(*t*)^)

8: Update $\mu :\mu^{\left(t+1\right)}=\{\begin{array}{ll} \text{min}\left(\rho \mu^{\left(t\right)},\mu_{\text{max}}\right) & if\mu^{\left(t\right)}\times \text{max}\left({\left\|Z^{\left(t+1\right)}-Z^{\left(t\right)}\right\|}_{F},{\left\|E^{\left(t+1\right)}-E^{\left(t\right)}\right\|}_{F}\right)<\varepsilon_{1}\\ \mu^{\left(t\right)} & Otherwise \end{array}$

9: Update t: *t* = *t* + 1

10: Until Convergence: $\frac{∥F-DZ^{\left(t\right)}-E^{\left(t\right)}{∥}_{F}}{{∥F∥}_{F}}<\varepsilon_{2}$

After the extraction of discriminative saliency, we divided the computed feature matrix into parts with the proposed model as the coefficients are *Z* = [*ZB*; *ZS*] over *D* = [*DB*, *DS*] and error *S*. Then, the salient region maps are computed using the background and foreground templates as:

$$\left\{ \begin{array}{c} S^{ST}={∥F-D_{ST}Z_{ST}∥}_{2}^{2}\\ S^{NT}={∥F-D_{NT}Z_{NT}∥}_{2}^{2} \end{array}\right.$$ (31)

where, *S*^*ST*^ and *S*^*NT*^ represents the reconstruction errors due to the salient and non-salient parts, respectively. We will get the final saliency results after merging the *S*^*ST*^ and *S*^*NT*^ maps using the following expression:

$$\begin{array}{c} S=S^{ST}\times (1-S^{NT}) \end{array}$$ (32)

**Fig 6:**
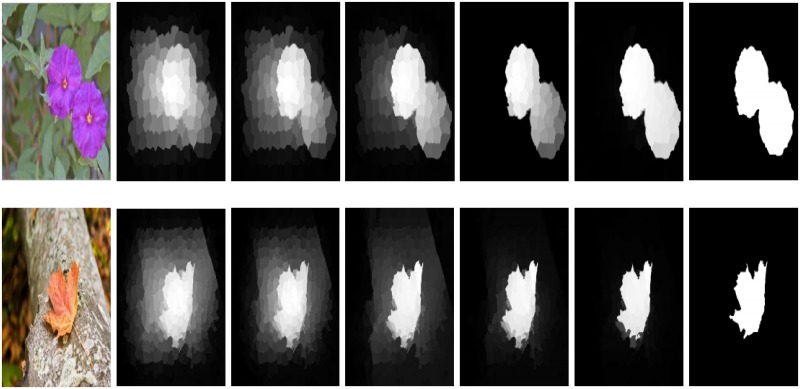
Our model complete transformation processes, the background part is diffuse as the iteration proceed, and at the end of iterations the process attains its ultimate saliency values. The results are similar but not identical to the original images and is therefore for illustrative purposes only.

We initialize the low-rank representation method [7], after the optimization procedure the values updated accordingly. We also note that the convergence of our method is fast and it takes only a small number of iteration for convergence as shown in Fig 6.

## 4 Experimental results and analysis

We analyzed and investigate our model on four largest benchmark datasets against existing methods. For performance assessment, four evaluation criteria are selected to completely analyze the proposed algorithm against the preceding schemes. In the next section, we discuss the details of selected benchmark datasets for performance evaluations.

### 4.1 Benchmark datasets

To analyze the computed saliency results, many datasets available that differ from each other in size, objects in the scene, image background like simple or complicated and the GT. We employ different datasets to assess the performance of our proposed algorithm. We assess our SRD model on four different standard datasets that are: 1) ASD [35], 2) ECSSD [36], 3) DUT-OMRON [5], 4) SED2 [37], and 5) MSRA [38]. We prefer these databases for the following reasons: 1) the background nature, 2) the complexity level, 3) the number of images, 4) the potential benchmark databases, and 5) the number of objects in the scene.

### 4.2 Preceding methods selected for comparison

Our SRD model is compared against fourteen state-of-the-art models. We first visually and then graphically compare to check and validate our framework. The schemes, we compare with our method are chosen due to these reasons: 1)citations, 2)recency, 3) computation complexity, and 4) variety. These schemes are: AC [39], FT [35], GB [9], HC [10], HS [36], MC [40], UFO [41], LC [42], SR [43], CH [44], GM [5], RB [45], RC [10], and DS [6]. All the source codes of above-defined approaches are easily accessible for public.

### 4.3 Evaluation metrics

Numerous techniques are applied to evaluate the concurrence between the obtained results and the GT. Before computing the evaluation metrics, the produced salient region maps should be changed in binary form to estimate the generated map. We also apply the adaptive threshold as discussed in [46], the thresholding is used to get the binary mask of salient region map *S*, that is calculated as:

$$\begin{array}{c} T_{h}=\frac{1}{w\times h}\sum_{a=1}^{h}\sum_{b=1}^{w}S\left(a,b\right) \end{array}$$ (33)

whereas, *w* and *h* represent the height and width of saliency map, respectively.

#### 4.3.1 Precision-Recall

The saliency map S is converted to the binary-mask M using the given ground truth T. The PR-curve is computed using this expression:

$$\begin{array}{c} Precision=\frac{\left|M\cap T\right|}{\left|M\right|},Recall=\frac{\left|T\cap M\right|}{\left|T\right|} \end{array}$$ (34)

#### 4.3.2 F-score

F-score is calculated using the Precision-Recall, the evaluation of the SRD is not complete without F-score. The F-score is computed using the following expression:

$$\begin{array}{c} F_{\nu }=\frac{(1+\nu^{2})\times Precision\times Recall}{\nu^{2}\times \left(Precision+Recall\right)} \end{array}$$ (35)

All of the compared method take the value of *ν* = 0.3. So, we have take the value of *ν* = 0.3 for a fair comparison.

#### 4.3.3 Receiver operating characteristics

The ROC-curve is obtained using the binary mask M with a fixed threshold as:

$$\begin{array}{c} TPR=\frac{|\bar{M}\cap T|}{|\bar{M}|},FPR=\frac{|M\cap \bar{T}|}{|\bar{T}|.} \end{array}$$ (36)

where, $\bar{T}$ is opposite of T and $\bar{M}$ is opposite of M. The ROC-curve is obtained through TPR and FPR with changing the value of the fixed threshold.

#### 4.3.4 Mean absolute error

To check the worth of SRD maps might have high significance as compared to binary mask. We also applied the MAE between the continuous SRD map S and the ground truth T, both are normalized in the range [0, 1]. The MAE value is defined as:

$$\begin{array}{c} MAE=\frac{1}{w\times h}\sum_{a=1}^{h}\sum_{b=1}^{w}\left|\bar{S}\left(a,b\right)-\bar{T}\left(a,b\right)\right| \end{array}$$ (37)

### 4.4 Impact of parameters and features on the performance of our model

In this section, we discuss the impact of different parameter on the performance of our proposed model. These parameters play a key role in obtaining the optimal results. These parameters are GR parameter, MMR parameter, and the sparsity parameter. We perform a series of experiments to adjust the values of these parameters. The details are discussed in the next subsection.

#### 4.4.1 Parameter settings

The performance of proposed model is severely affected by the GR parameter, MMR parameter, the sparsity parameter and the number of superpixels. Therefore, for the optimal results the values of these key parameters are adjusted as *α*_1_ = 1, *α*_2_ = 0.1, *α*_3_ = 10^5^, and *N* = 300.

#### 4.4.2 Impact of GR parameter

**Fig 7:**
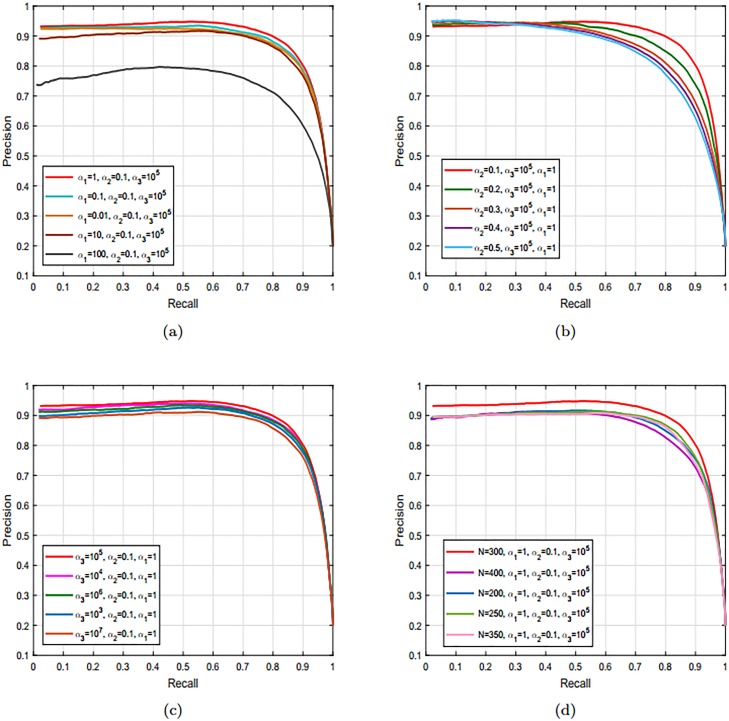
Impact of different key parameters on the performance of our designed framework using the MSRA dataset [38]. (a) A series of PR-curves obtained with varying the values GR parameter. (b) The PR-curve obtained using the different values of the sparsity parameter in the range starting from {0.1} to {0.5}. (c) The impact of MMR parameter with varying its values in the range between {10^3^, 10^7^}. (d) The impact of the number of superpixels on the performance of the proposed model.

Normally, this parameter is theoretically utilized to penalize the discontinuities and geometrical features in representation coefficients. We also perform a series of simulation with varying the values Laplacian parameter. The results revealed that with the higher values the geometrical structures of the salient objects are lost. While, with the lower values of the Laplacian parameter, structural and geometrical features are preserved as revealed in Fig 7(a). The Laplacian regularization parameter saves the structural and geometrical features and means discrepancy parameter reduces the distribution divergence and connections among similar regions.

#### 4.4.3 Effect of sparsity factor

To check the impact of the sparsity parameter, we use different values to run the simulation for the sparsity parameter in the range starting from {0.1} to {0.5}. Generally, the sparsity parameter is engaged to keep away from deterioration and over-fitting. From the simulation, we note that with the smaller value of *α*_2_ or as we move toward the zero a few numbers of iteration are required. In the contrast, if the value of sparsity parameter moves in the direction of infinity the computed saliency maps not remain significant due to the supremacy of the sparsity as demonstrated in Fig 7(b).

#### 4.4.4 Impact of MMR parameter

To check the impact of MMR parameter, we perform the simulation with varying its values in the range between {10^3^, 10^7^}. From the experimental results shown in Fig 7(c), we found that with the larger value of *α*_3_ the distribution connection between the regions is assured while the sparsity is badly affected. In the contrast, if we choose the smaller value for the MMR parameter the geometrical structure of the salient object and the sparsity is secure while the distribution connection between the regions is lost. In both of the cases, the sparsity of the approach is also not robust. For the ease and efficiency, we select the value of *α*_3_ = 10^5^.

#### 4.4.5 Impact of number of superpixels

We also check the impact of the number of superpixels on the performance of the proposed model as shown in Fig 7(d). The numbers of superpixels increase the performance at a fixed value. The performance is unaffected by some values at a certain level. However, we selected the value of *N* = 300 and at this value, the proposed model performs well.

#### 4.4.6 Effect of visual and textural feature on the performance of our proposed model

**Fig 8:**
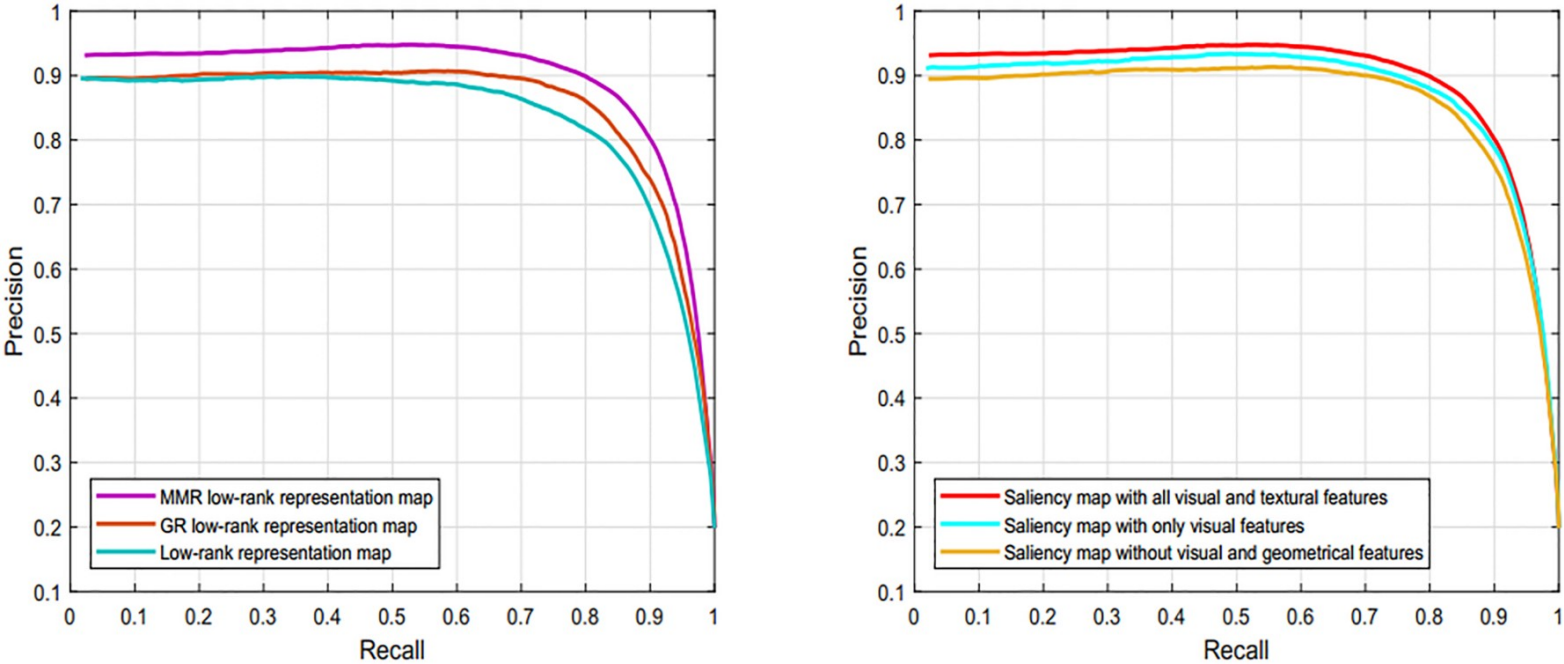
The soundness and efficiency of proposed model through the visual features, textural features and different regularization terms using the MSRA dataset [38].

We perform simulations and used PR curve to check the effect of features on the efficiency of our model. The comparisons between the different features of our model are shown in Fig 8(a). It is clear from the revealed results that the PR-curve only with textural features is slightly lower than the other two curves and the middle curve is obtained using the visual features. In both the cases, the obtained results are not accurate and losing a lot of image information. Consequently, both the curves are below than the curves contain both the visual and textural features. Hence, the visual and textural features are necessary to obtain the final saliency results and without including this information the precise results cannot be calculated.

#### 4.4.7 Effect of regularization terms on the final saliency

We also analyze the impact of regularization terms like Laplacian regularization and mean discrepancy term on the final saliency map. By regularizing the low-rank representation through these terms can save the similarity as well as the locality of the regions. Here, we exploited the Laplacian term for representation coefficients and a MMRT for representation errors. By using, these terms the representation coefficients and representation errors of similar regions contain similar saliency values when sparsely encoded with the discriminative dictionary. We can note from the Fig 8(b) the remarkable improvements in the final saliency results are obtained by adding these regularization terms.

### 4.5 Visual analysis

**Fig 9:**
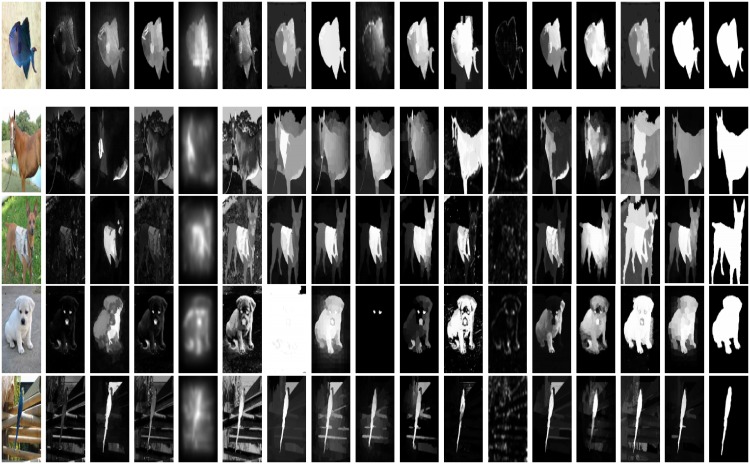
Comparison of proposed framework visually with the state-of-the-art methods on the MASRA dataset [38]. The SRD results are arranged as: II, AC [39], CH [44], FT [35], GB [9], HC [10], HS [36], MC [40], GM [5], RB [45], RC [10], SR [43], UF [41], DS [6], LC [42], SRD results of our model, and GT.

We analyze our model visually with the current schemes like: AC [39], CH [44], FT [35], GB [9], HC [10], HS [36], MC [40], GM [5], RB [45], RC [10], SR [43], UF [41], DS [6], and LC [42] on the MSRA [38] dataset. The reason for selecting this dataset is that MSRA dataset contains almost 10,000 natural images. The images in this dataset contain all type of variations like the plane and complicated background, patterned and textured background, simple and diversify background and the center and side salient objects images. We selected the images of different type like the salient object touching the boundary (the second row of Fig 9), the contrast between foreground and background is less (the fourth row of Fig 9), and background is diversified or complicated as demonstrated in the third and fifth row of Fig 9. The performance of LC [42], DS [6], HS [36], and [40] is satisfactory in all these images. LC [42] and MC [40] perform well for centered salient objects but as the salient objects touch the any side of the image. LC [42] and MC [40] lose accuracy and start adding the background part with the salient objects. MC is not good in dealing with cluttered images. DS [6] perform well, however, lost the salient objects part when the salient object pops up near the image boundaries due to incompetent background dictionary. While the HS [36] totally loss the salient objects in the case of less contrast between the background and foreground as shown in the Fig 9. We can note that in all the type of images the proposed performance remains consistent and does not lose any image information as compared to the existing schemes.

#### 4.5.1 ASD dataset

**Fig 10:**
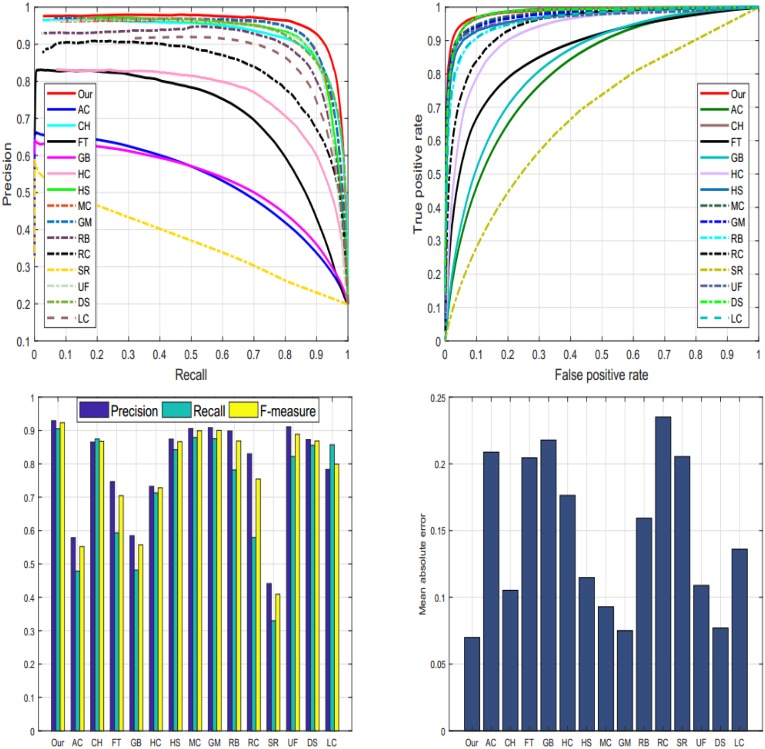
The graphical comparison of our method against the current schemes like: AC [39], CH [44], FT [35], GB [9], HC [10], HS [36], MC [40], GM [5], RB [45], RC [10], SR [43], UF [41], DS [6], LC [42], and our method on the ASD dataset [35].

We assess the performance of our SRD scheme with state-of-the-art methods on the ASD database [35] as revealed in Fig 10. The reason for selecting ASD database is to investigate the behavior of our scheme with images having different complexity levels and diversified pattern. We examine the proposed method against fourteen most well-known SRD schemes such as: AC [39], CH [44], FT [35], GB [9], HC [10], HS [36], MC [40], GM [5], RB [45], RC [10], SR [43], UF [41], DS [6], and LC [42]. The above-discussed metrics are engaged for evaluation of our method, we found that the proposed model performs against the chosen methods with the higher accuracy as revealed through the Tables 1, 2 and 3. However, HC, DS, and MC as well performed persuasively. On this dataset, we note that our method remains extremely reliable and accurate in dealing with the salient objects.

**Table 1:** Comparison of F-score of the proposed model with current approaches.

MODEL	AC	CH	FT	GB	HC	HS	MC	GM	RB	RC	SR	UF	DS	LC	OUR
ECSSD	0.41	0.684	0.431	0.583	0.441	0.659	0.704	0.712	0.68	0.691	0.385	0.654	0.699	0.396	0.738
SED2	0.675	0.688	0.685	0.529	0.655	0.776	0.766	0.749	0.815	0.729	0.500	0.721	0.747	0.644	0.802
D-OM	0.343	0.566	0.388	0.448	0.36	0.565	0.583	0.571	0.58	0.558	0.303	0.521	0.579	0.313	0.699
ASD	0.566	0.864	0.701	0.587	0.763	0.852	0.900	0.895	0.889	0.778	0.411	0.896	0.884	0.802	0.920

**Table 2:** Comparison of AUC of the proposed model with current approaches.

MODEL	AC	CH	FT	GB	HC	HS	MC	GM	RB	RC	SR	UF	DS	LC	OUR
**ECSSD**	0.668	0.903	0.661	0.865	0.704	0.883	0.91	0.889	0.894	0.892	0.633	0.875	0.914	0.627	0.907
**SED2**	0.831	0.831	0.82	0.839	0.88	0.858	0.877	0.862	0.899	0.852	0.769	0.845	0.915	0.827	0.861
**D-OM**	0.721	0.89	0.682	0.857	0.733	0.86	0.887	0.853	0.894	0.859	0.688	0.839	0.899	0.654	0.895
**ASD**	0.756	0.952	0.79	0.902	0.867	0.933	0.951	0.944	0.955	0.936	0.736	0.938	0.959	0.771	0.953

**Table 3:** Comparison of MAE of the proposed model with current approaches.

MODEL	AC	CH	FT	GB	HC	HS	MC	GM	RB	RC	SR	UF	DS	LC	OUR
**ECSSD**	0.352	0.249	0.355	0.310	0.360	0.268	0.254	0.239	0.230	0.246	0.312	0.267	0.225	0.339	0.222
**SED2**	0.206	0.168	0.206	0.242	0.193	0.157	0.182	0.163	0.13	0.148	0.22	0.18	0.14	0.204	0.145
**D-OM**	0.19	0.152	0.25	0.24	0.31	0.227	0.186	0.189	0.144	0.189	0.181	0.272	0.139	0.246	0.125
**ASD**	0.227	0.112	0.213	0.232	0.175	0.139	0.095	0.090	0.085	0.117	0.212	0.125	0.080	0.163	0.070

#### 4.5.2 DUT-OMRON dataset

**Fig 11:**
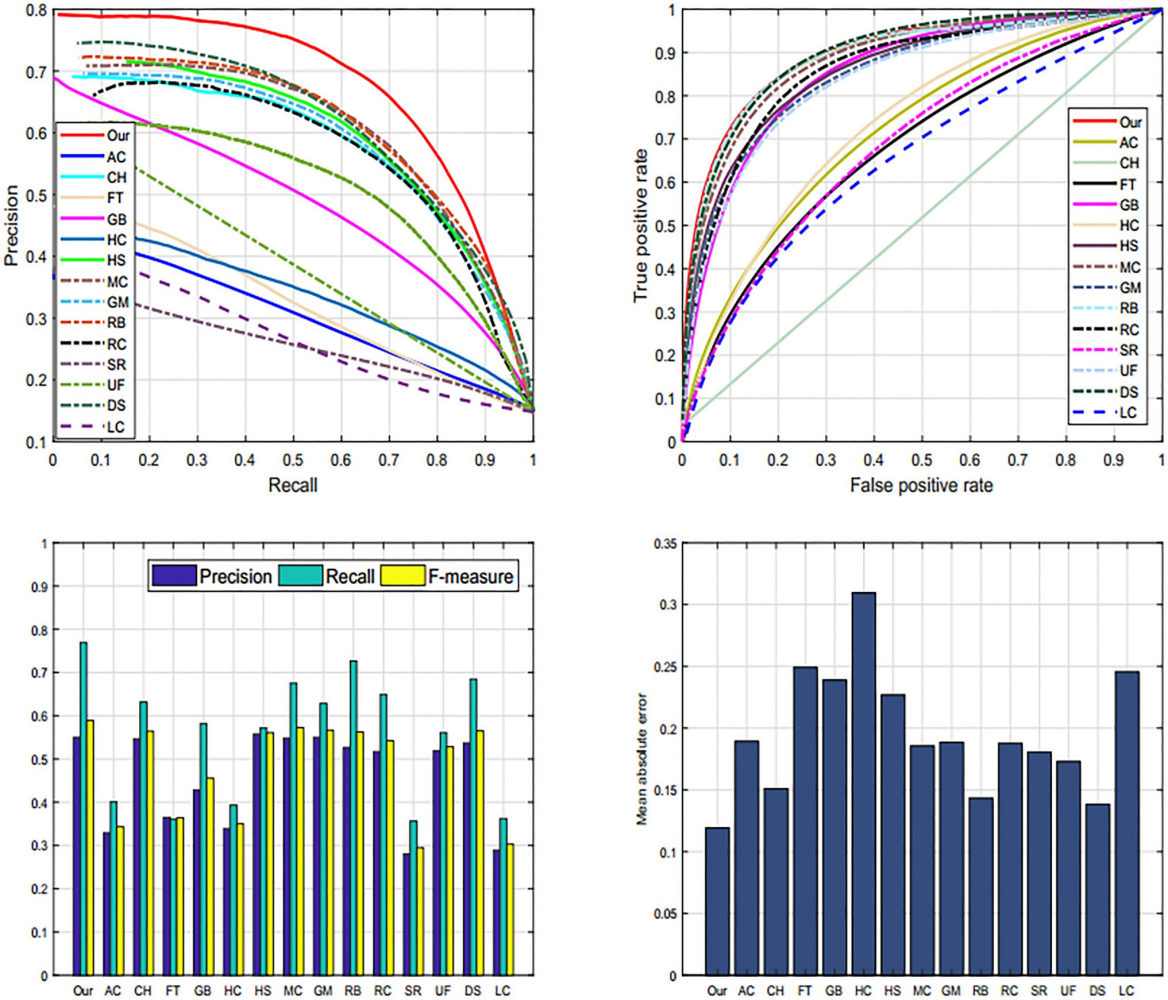
Graphical comparison of our method with current schemes such as: AC [39], CHM [44], FT [35], GB [9], HC [10], HS [36], MC [40], GM [5], RB [45], RC [10], SR [43], UF [41], DS [6], LC [42], and our proposed model on the DUT-OMRON database [5].

We also use DUT-OMRON dataset [5] to analyze the performance of our proposed approach. The motive for selecting DUT-OMRON database [5] is that it contains a large number of images with different complexity levels of the background. Therefore, we use this database to evaluate our approach. We verify the performance of our proposed model graphically PR and ROC-curves. The resulting graphs are illustrated in Fig 11. Nevertheless, DS [6], GM [5], and RB [45] also demonstrate persuasive results as described in Tables 1, 2 and 3. We notice from our analysis that our approach is more effective in highlighting the salient objects than the other discussed methods.

#### 4.5.3 ECSSD dataset

**Fig 12:**
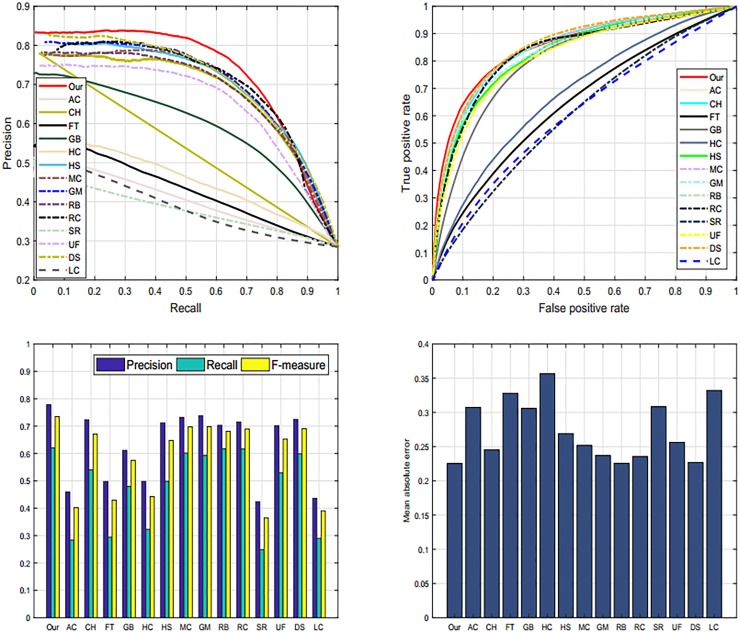
The graphical comparison of proposed model PR-curve, ROC-curve, F-measure, and MAE with existing approaches like: AC [39], CH [44], FT [35], GB [9], HC [10], HS [36], MC [40], GM [5], RB [45], RC [10], SR [43], UF [41], DS [6], and LC [42] on the ECSSD database [36].

Moreover, we as well engaged ECSSD dataset [36] to assess and certify our SRD mechanism visually and graphically. ECSSD database contains more natural images with a diversified pattern for both foreground and background. The reason for selecting ECSSD database is to investigate the behavior of our scheme with images having different complexity levels and diversified pattern. We examine the proposed method against fourteen most well-known SRD schemes such as: AC [39], CH [44], FT [35], GB [9], HC [10], HS [36], MC [40], GM [5], RB [45], RC [10], SR [43], UF [41], DS [6], and LC [42] on the ECSSD database to declare the strength of our algorithm. We pick four different criteria, which are mainly used in the literature to assess the performance of SRD methods. These criteria are PR-curve, ROC curve, F-score, and MAE to check the performance of our proposed approach. From the series of experiments as given in Tables 1, 2 and 3, we found that our proposed method achieves very good results as compared to above-defined approaches. On the other hand, DS [6], GM [5], and RC [22] as well accomplished fine results on all four SRD metrics. Our approach remains unswerving in all defined evaluation measures and demonstrates significant performance as shown in Fig 12.

#### 4.5.4 SED2 dataset

**Fig 13:**
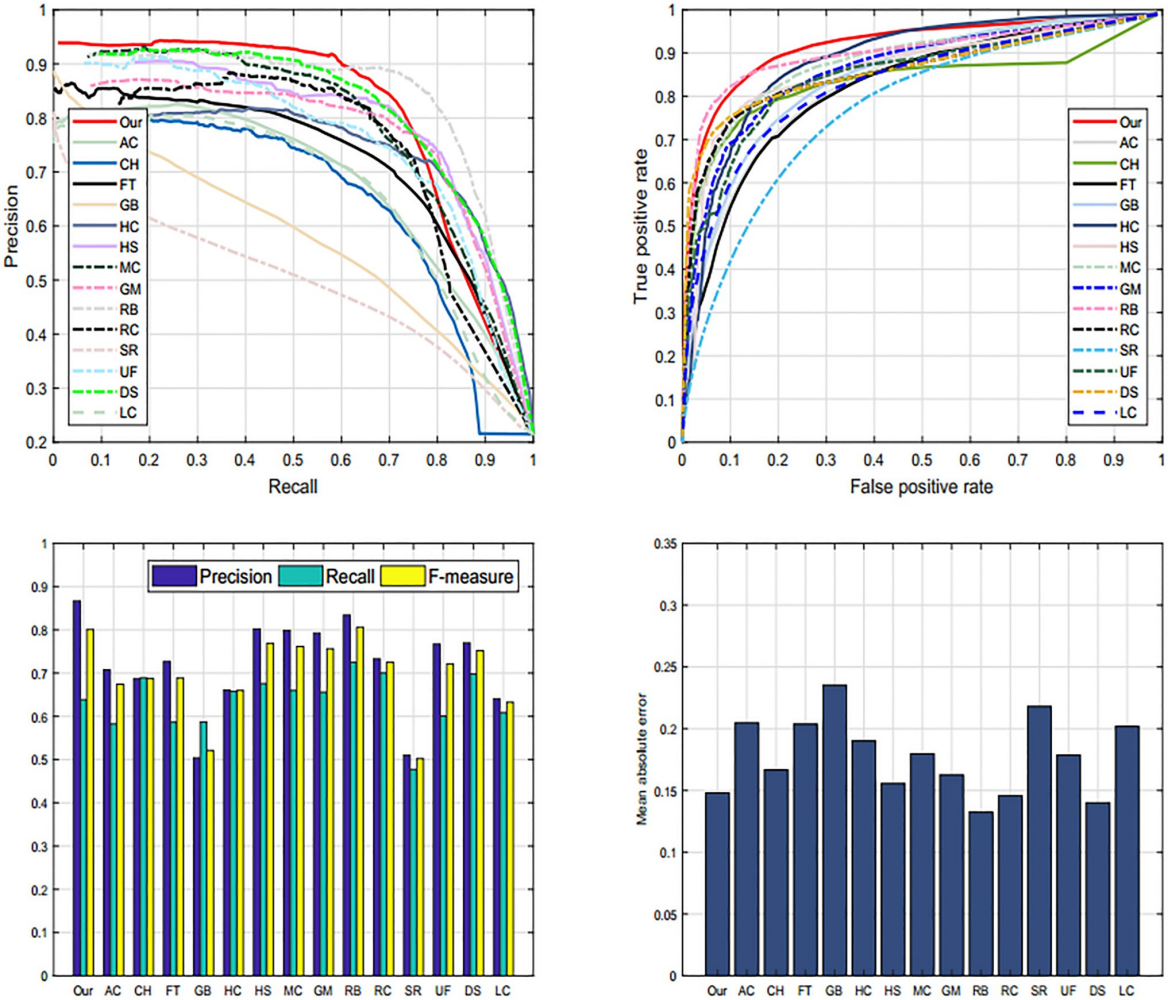
The graphical comparison of proposed model with existing models like: AC [39], CH [44], FT [35], GB [9], HC [10], HS [36], MC [40], GM [5], RB [45], RC [10], SR [43], UF [41], DS [6], and LC [42] on the SED2 dataset.

Additionally, we employed SED2 dataset [37] to evaluate and validate the proposed method graphically. The motive for electing SED2 database is to assess the performance of our scheme through an image with two objects. We analyze and compare the proposed method against fourteen most famous state-of-the-art approaches such as: AC [39], CH [44], FT [35], GB [9], HC [10], HS [36], MC [40], GM [5], RB [45], RC [10], SR [43], UF [41], DS [6], and LC [42] on SED2 database to assure the validity of our algorithm. We choose four different criteria like PR-curve, ROC curve, F-measure, and MAE to estimate the strengths and bounds of our SRD approach as revealed in Tables 1, 2 and 3. Our SRD model remains very consistent in all of the evaluation measures and shows a remarkable performance as illustrated in Fig 13.

### 4.6 Limitation

**Fig 14:**
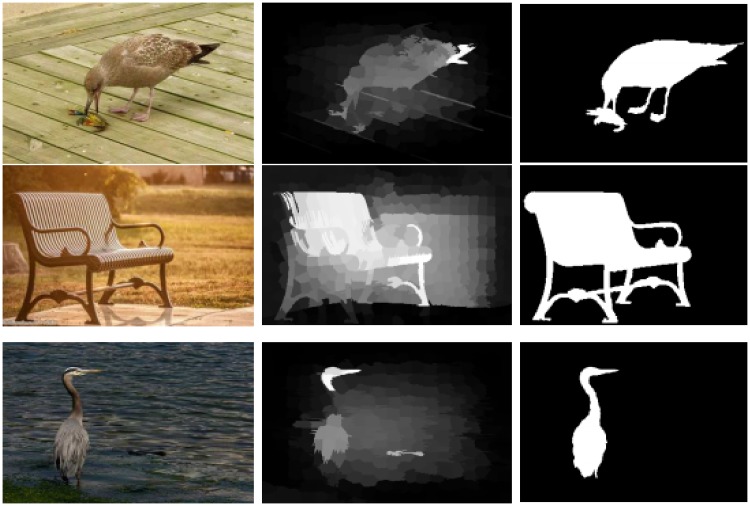
Some cases in which the designed method results are not satisfactory. The results are similar but not identical to the original images and is therefore for illustrative purposes only.

Our designed model performs against the current salient regions detection methods. On the other hand, in some typical images, our model computed results are poor as demonstrated in the Fig 14. In these typical images, the salient object is almost in the same color as the background. During the SRD, some background parts are incorrectly considered as a foreground and embed with the salient object leading to poor performance. This problem is famous in object detection when the contrast is very the objects are not detected properly.

### 4.7 Saliency cut

**Fig 15:**
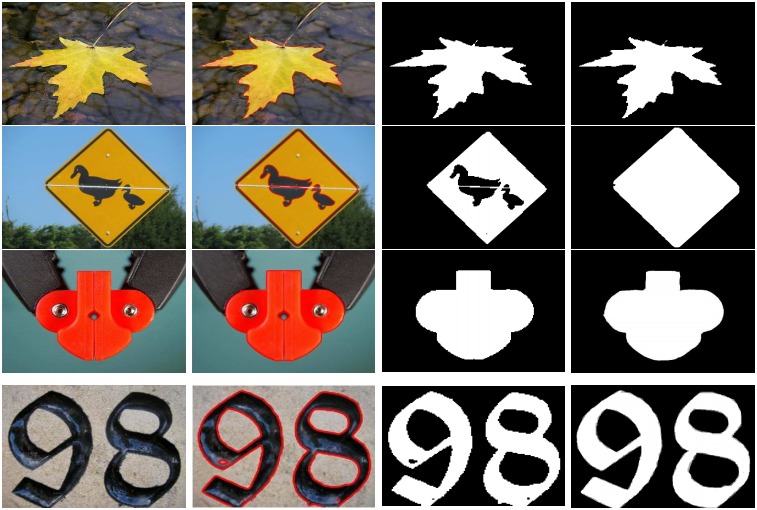
The segmentation and saliency cut results generated through the proposed model. The results are arranged as: given image, segmented results, saliency cut results, and the GT. The results are similar but not identical to the original images and is therefore for illustrative purposes only.

In the current literature, different methods employ the shape prior to segment the salient object, which is later utilized to produce the saliency cut. Some others used a rectangular locale to capture the salient object and then this captured region is converted to a fuzzy region. Both of the above-mentioned methods use the different strategies to compute the saliency cut. The proposed method also segments the image first and then produces a precise saliency cut, we use different datasets and found that the proposed model produces quite promising saliency cut results as demonstrated in Fig 15.

## 5 Conclusion

In this paper, we propose a novel SRD method through salient and non-salient dictionaries. Initially, a new feature space is constructed by concatenating four feature spaces like CIELab, RGB, HOG, and LBP. Then, we combine a boundary metric, candidate objectness metric and a candidate distance metric to compute a low-level saliency map. After that, we extract a salient template and a non-salient dictionary from that low-level saliency. We regularize the low-rank representation through GRT that saves the structural and geometrical features and using a MMRT that reduces the distribution divergence and connections among similar regions. The proposed model is tested against over a dozen latest SRD method using four evaluation metrics. The proposed model remains persistent in all the tests and outperformed against the selected models with higher precision.
